# Comparative Transcriptome Analysis Reveals the Role of the FST Gene in Goose Muscle Development

**DOI:** 10.3390/ani15203009

**Published:** 2025-10-16

**Authors:** Cui Wang, Yi Liu, Mingxia Li, Yunzhou Yang, Jiuli Dai, Shufang Chen, Huiying Wang, Daqian He

**Affiliations:** 1 Institute of Animal Science and Veterinary Medicine, Shanghai Academy of Agricultural Sciences, Shanghai 201106, China; cuiwang518@saas.sh.cn (C.W.); liuyi20031194@163.com (Y.L.); kobebryant198687@163.com (Y.Y.); 2 Academy of Life Sciences and Technology, Tongji University, Shanghai 200092, China; 2110839@tongji.edu.cn; 3 NingBo Academy of Agricultural Sciences, Ningbo 315040, China; 13858357201@163.com (J.D.); 13606780161@163.com (S.C.)

**Keywords:** *gFST*, expression profile, SMSCs, overexpression, RNA-seq

## Abstract

**Simple Summary:**

Muscle growth has a significant impact on the economic value of goose meat. In this study, we investigated the follistatin (FST) gene in Zhedong White geese and identified two distinct transcript variants. We found that *FST* gene activity changes during goose development and in muscle cells. Notably, overexpressing *FST* in goose muscle cells downregulated key genes involved in myogenesis and upregulated genes related to fat metabolism. These results indicate that *FST* plays a critical role in shifting the balance from muscle differentiation toward fat deposition in geese. Our findings provide valuable insights into the mechanisms underlying muscle development and meat quality in poultry.

**Abstract:**

Muscle growth is a critical determinant of meat yield and quality in livestock. Although follistatin (FST) is recognized as a key regulator of skeletal muscle development and fat metabolism, its specific function in geese remains largely unexplored. In this study, we identified two transcript variants of goose FST (*gFST*) in Zhedong White geese: gFST-X1 (1125 bp), encoding a 343-amino acid protein with a 28-amino acid signal peptide and four conserved domains, and gFST-X2, which contains a 243 bp insertion within the gFST-X1 transcript. RT-qPCR analysis revealed that *gFST* mRNA expression varied across tissues from female embryos (25 days), adults (70 days), and laying geese (270 days), as well as in skeletal muscle satellite cells (SMSCs) at embryonic day 16 (E16d). Overexpression of gFST in SMSCs resulted in 3596 differentially expressed genes (DEGs), including 2247 upregulated and 1349 downregulated genes (*padj* < 0.01). Key stemness markers (*PAX7*, *PAX3*) and myogenic regulators (*MYOG*, *MYOD*, *MYF5*) were significantly downregulated, whereas genes associated with lipid metabolism (*PPARG*, *FABP5*, *ACSL5*) and myosin-related processes (*MYO1D*, *MYO1F*, *MYO1E*) were markedly upregulated (*padj* < 0.01). Functional enrichment analysis linked these DEGs to the TGF-β, PPAR signaling, fatty acid metabolism, and Notch signaling pathways. These transcriptomic findings were further validated by qRT-PCR. Collectively, our results demonstrate the dual regulatory role of *gFST* in skeletal muscle development and provide new mechanistic insights into muscle development in geese.

## 1. Introduction

Follistatin (FST) is a glycosylated single-chain protein that acts as a potent antagonist of cytokines in the transforming growth factor-β (TGF-β) superfamily, including activins, myostatin, and bone morphogenetic proteins (BMPs) [1,2]. By binding and neutralizing these ligands, *FST* primarily exerts its biological effects through the liberation of key anabolic and metabolic pathways from their inhibitory constraints [2]. In mammals, *FST* is recognized as a critical regulator of diverse biological processes, notably enhancing muscle growth, suppressing adipose tissue deposition, and improving metabolic homeostasis and insulin sensitivity [3,4,5]. Similarly, in poultry, due to its strong inhibitory effect on myostatin—a well-known negative regulator of muscle mass—*FST* is considered a promising candidate gene for improving meat yield [6].

Initially isolated from follicular fluid and characterized as a follicle-stimulating hormone (FSH)-suppressing protein [7,8], *FST* gene has been extensively investigated in various livestock species. In transgenic pigs, *FST* expression leads to enhanced skeletal muscle growth, reduced fat deposition, and improved metabolic profiles [9]. In sheep, polymorphisms in the *FST* gene have been associated with wool quality traits [10]. In poultry, studies in ducks have demonstrate that *FST* promotes muscle development through the activation of satellite cells in vivo [11,12], whereas in chickens, its expression varies dynamically during development, and specific polymorphisms are significantly correlated with growth-related traits [13]. Together, these findings highlight the conserved role of *FST* as a key regulator of skeletal muscle development and lipid metabolism, supporting its potential as a target in molecular breeding programs.

Skeletal muscle, the primary edible tissue in meat-producing animals, is a major determinant of production efficiency and economic value [14]. Its development originates from myogenic precursor cells (myoblasts) that proliferate and differentiate into myotubes, ultimately forming mature muscle fibers [15]. In both avian and mammalian species, the total number of muscle fibers is largely determined during embryogenesis. Postnatal muscle growth is therefore primarily driven by the hypertrophy of these existing fibers, a process mediated by skeletal muscle satellite cells (SMSCs). As the principal stem cells responsible for muscle growth, maintenance, and regeneration [16,17,18], SMSCs have become a central model for investigating the molecular mechanisms that regulate skeletal muscle development.

In poultry, SMSCs isolated from mid-incubation embryos (e.g., E12-E18) exhibit robust proliferation and differentiation capacities, and are commonly purified using the differential plating method [19,20,21,22]. Recent studies have revealed complex regulatory networks, including various non-coding RNAs, that control chicken SMSC growth by modulating critical signaling pathways [23,24,25,26,27,28]. In geese, our previous research defined distinct developmental stages of SMSCs and delineated stage-specific molecular signatures during differentiation [22]. Intriguingly, we observed a marked downregulation of the goose follistatin (*gFST*) gene at the onset of differentiation, suggesting its potential regulatory role. However, the specific biological functions of *gFST* in this context remain largely unexplored.

Geese are economically significant poultry species, primarily raised for meat production. As the world’s leading producer, China contributes more than 2.5 million tons of goose meat annually, accounting for over 90% of global output. In this study, we aimed to characterize the *gFST* gene and elucidate its functional role in skeletal muscle development. We cloned and identified *gFST* transcript variants and analyzed their mRNA expression pattern across various tissues and developmental stages. Additionally, using comparative transcriptomics, we explored the global changes in gene expression and key regulatory networks influenced by *gFST* overexpression in goose SMSCs. The results are expected to clarify the mechanisms by which *gFST* governs skeletal muscle growth, providing a valuable theoretical basis for the genetic improvement of goose breeding.

## 2. Materials and Methods

### 2.1. Animals and Sample Collection

Goose embryos at 16 days (E16d) and 25 days (E25d) of development (n = 20 per stage), along with female adult geese at 70 days (A70d; n = 4) and laying geese at 270 days (L270d; n = 4), were provided by the Wenjie Goose Breeding Department of Xiangshan Co. Ltd. (Ningbo, China). The selection of these developmental stages was designed to capture key transitions in skeletal muscle development, spanning embryonic myogenesis, sexual maturation, and the laying period. Embryos were incubated in a standard commercial incubator (Zhonglian, China). Post-hatch geese were reared under standard commercial management conditions with access to open ground and a swimming pool. The sex of E16d and E25d embryos was determined using CHD1-F/R primers (Table S1). Leg muscle tissue from A70d geese was used to clone the *gFST* cDNA sequence. Various tissues—including heart, liver, spleen, lung, kidney, breast muscle, leg muscle, brain, skin, muscular stomach, hypothalamus, pituitary, and ovary—were collected from three key developmental stages (E25d, A70d, and L270d). All samples were immediately snap-frozen in liquid nitrogen and stored at −80 °C for subsequent RNA isolation. All animal procedures were approved by the Institutional Animal Care and Use Committee of Shanghai Academy of Agricultural Sciences (License number: SAAS-SL-2023021).

### 2.2. Molecular Cloning of gFST cDNA

Total RNA was extracted using Trizol Reagent (Invitrogen, Waltham, MA, USA) and treated with RNase-free DNaseI (TaKaRa, Dalian, China) to remove genomic DNA contamination. First-strand cDNA was then synthesized from 1 µg of total RNA using the PrimeScript^TM^ RT reagent Kit with gDNA Eraser (TaKaRa, Dalian, China). The cDNA was diluted to a work concentration of 100–300 ng/µL and stored at −20 °C for subsequent qPCR analysis. To amplify the *gFST* coding sequence, a pair of primers (FST-F/R, Table S1) was designed based on conserved regions of the *FST* gene in chicken (NM_205200.2) and duck (XM_027446660.2). The PCR program included: 95 °C for 5 min; 38 cycles of 95 °C for 30 s, primer annealing for 30 s, and 72 °C for 90 s; followed by a final extension at 72 °C for 5 min. The amplified fragments were subsequently cloned into the PEASY-T1 cloning vector (TransGen, Beijing, China) and sequenced by Sangon Biotech (Shanghai, China). Sequence assembly, analysis, and alignment were conducted using DNAMAN 9.0, SeqMan (DNAstar, Madison, WI, USA), CLUSTALW, and ESPript 3.0 software.

### 2.3. Tissue Expression Pattern Analysis of gFST mRNA

qPCR was performed in a 20 uL reaction mixture containing 10 uL of 2× TB Green Premix Ex Taq II (Tli RNaseH Plus) (Bio-Rad, Hercules, CA, USA), 2 μL of cDNA template, 0.5 μL of each primer (10 μM), and 7 μL of nuclease-free water. Amplification was conducted on a 384-well C1000 Touch^TM^ Thermal Cycler (Bio-Rad, Hercules, CA, USA) using the following program: an initial denaturation at 95 °C for 2 min, then 40 cycles of 95 °C for 5 s and 60 °C for 30 s. The primers FST-QF/QR and β-actin-F/R (Table S1) were used to quantify *FST* mRNA levels in tissues from E25d, A70d, and L270d geese. Reaction specificity was verified by melting curve analysis. Each sample was run in triplicate, and relative gene expression levels were calculated using the comparative 2^−ΔΔCt^ method.

### 2.4. Goose SMSC Isolation, Purification and Expression Analysis

Based on our previous finding that SMSCs isolated from E16d geese exhibit strong proliferative and differentiative capacities, leg muscle tissues were collected from female E16d embryos (n = 8) for SMSC isolation using a previously established goose-specific protocol [22]. Following sex identification, the female embryos were disinfected with ethanol. The leg muscles were carefully dissected, and any blood vessels, adipose tissue, and connective tissue were removed. The muscle tissues were then minced into a paste and digested for 50 min at 37 °C with 2 mg/mL Dispase II (Roche, Basel, Switzerland) and 4 mg/mL Collagenase II (Gibco, Grand Island, NY, USA) in high-glucose DMEM (Corning, Grand Island, NY, USA). Digestion was terminated by adding high-glucose DMEM supplemented with 10% FBS (Lonsera, Ciudad de la Costa, Uruguay, South America). The resulting suspension was filtered through a 70 µm mesh sieve and centrifuged at 350× *g* for 8 min at room temperature. Red blood cells were subsequently removed using ACK lysis buffer (Gibco, Grand Island, NY, USA).

The cell pellet was resuspended in DMEM/F12 medium (Gibco, Grand Island, NY, USA) supplemented with 10% FBS, 1% penicillin–streptomycin (PS; Gibco, Invitrogen, Waltham, MA, USA), and 5 ng/mL basic fibroblast growth factor (bFGF; RD Systems). The cells were then cultured in an incubator (Thermo Forma, Thermo Fisher Scientific Inc., Waltham, MA, USA) at 37 °C with 5% CO_2_. To separate SMSCs from fibroblasts and adipocytes, which adhere more rapidly, the culture supernatant containing non-adherent cells was transferred to a new Petri dish after one hour. This differential adhesion step was repeated twice to further enrich the SMSC population. Finally, SMSCs were harvested every 12 h for subsequent expression analysis.

Immunofluorescence staining was performed to identify the isolated SMSCs. Briefly, cells cultured in a 6-well plate were washed three times with PBS and fixed with 4% paraformaldehyde for 20 min. After three additional PBS washes, the cells were permeabilized with 0.25% Triton X-100 in PBS for 10 min and then blocked with a solution containing 2% BSA and 0.05% Triton X-100 in PBS for 60 min at room temperature. Subsequently, the cells were incubated overnight at 4 °C with a primary antibody against Pax7 (Abcam, Cambridge, UK). Following PBS washes to remove unbound primary antibody, the cells were incubated with a fluorescently conjugated secondary antibody (1:2000 dilution; Thermo Fisher Scientific Inc., Waltham, MA, USA) for 1 h at room temperature protected from light. After further washing, nuclei were counterstained with 1× DAPI (10 µg/mL in PBS) for 20 min in the dark. Finally, fluorescent images were acquired using a fluorescence microscope (OLYMPUS, Tokyo, Japan), and the percentage of Pax7-positive cells was quantified using Aipathwell v2 software (Servicebio, Wuhan, China).

### 2.5. Overexpression Vector Construction, Cell Transduction, and FACS Analysis

The coding sequence of *gFST* was cloned into the pKLV2-U6gRNA5 (Empty)-PGKmCherry2AGFP plasmid (Addgene, #67981) via NotI and EcoRI restriction sites using the ClonExpressII One Step Cloning Kit (Vazyme, C112).

For lentivirus production, 293FT cells at 70–80% confluency in 10 cm dishes were transfected with a mixture of 4 μg psPAX2, 2 μg pMD2.G, and 5 μg of the FST-X1 transfer plasmid complexed with 27.5 μL of liposomal transfection reagent (Yeasen, Shanghai, China) in 500 μL Opti-MEM (Gibco, Invitrogen, Waltham, MA, USA). The mixture was incubated at room temperature for 20 min to allow DNA–lipid complex formation before being applied to the cells. The viral supernatant was harvested 72 h post-transfection, filtered through a 0.45 μm membrane, and either used directly for transduction or stored at −80 °C.

For transduction, purified goose SMSCs were seeded in 6-well plates and infected at 80–90% confluency by replacing the culture medium with 1 mL of viral supernatant. At three days post-transduction, approximately 1 × 10^6^ cells were harvested, washed with FACS buffer (PBS with 2% FBS), resuspended in 300 μL of the same buffer, and passed through a 40 μm cell strainer. FACS was then performed to isolate the tdTomato-positive cell population for subsequent RNA sequencing.

### 2.6. RNA Extraction, Library Preparation and Transcriptome Analysis

Total RNA was extracted from both positive and negative cell populations using Trizol Reagent (Invitrogen, Waltham, MA, USA). RNA concentration was measured with a NanoDrop NC2000 spectrophotometer (Thermo Fisher Scientific Inc., Waltham, MA, USA), and RNA integrity was confirmed by agarose gel electrophoresis. Following quality assessment, 3 µg of high-quality total RNA was used as input for cDNA library construction with the NEBNext Ultra II RNA Library Prep Kit for Illumina (New England Biolabs Inc., Ipswich, MA, USA), according to the manufacturer’s protocol. The prepared libraries were sequenced on an Illumina NovaSeq 6000 platform (Personal Biotech, Shanghai, China).

Raw sequencing reads were processed with fastp (v0.22.0) to ensure data quality, which involved trimming 3′ adapter sequences and removing reads with an average quality score below Q20. The cleaned reads were then aligned to the reference genome (GCF_002166845.1) using HISAT2 (v2.1.0) [29]. Gene expression levels were quantified with HTSeq (v0.9.1) [30], and read counts were normalized using the FPKM method [31].

Differential expression analysis was performed using DESeq (v1.38.3) [32]. Genes with |log_2_FoldChange| > 1 and an adjusted *p*-value < 0.05 were considered differentially expressed. Bidirectional hierarchical clustering of these differentially expressed genes (DEGs) was conducted with the Pheatmap R package (v1.0.12) [33], grouping genes into clusters based on similar expression profiles. Enrichment maps were generated to visualize significantly enriched functional terms within each cluster.

To interpret the biological roles of the DEGs, Gene Ontology (GO) enrichment analysis was carried out using topGO (v2.50.0) [34], with terms considered significantly enriched at an adjusted *p*-value < 0.05. KEGG pathway enrichment analysis was performed using clusterProfiler (v4.6.0) [35], retaining pathways with an adjusted *p*-value < 0.05. Additionally, Gene Set Enrichment Analysis (GSEA) was conducted using GSEA software (v4.1.0) [36], and an enrichment pathway map was generated from the results.

### 2.7. Verification of DEGs by qRT-PCR

To validate the transcriptome sequencing results, we selected eight DEGs that are significantly differentially expressed and functionally relevant to skeletal muscle cell development. Gene-specific primers were designed using Oligo 6.0 software based on reference sequences from the NCBI database; the details of these genes and their corresponding primers are listed in Table S1. qRT-PCR was performed using TB Green Premix Ex Taq II (Tli RNaseH Plus) (Bio-Rad, Hercules, CA, USA) on a 384-well C1000 Touch^TM^ Thermal Cycler (Bio-Rad, Hercules, CA, USA). Following amplification, a melting curve analysis was conducted to confirm the specificity of the PCR products.

### 2.8. Statistical Analyses

Gene expression levels were quantified using the comparative Ct (2^−ΔΔCt^) method, with goose *β-actin* serving as the endogenous reference gene for normalization. Data are expressed as mean ± standard error of the mean (SEM). Statistical significance was determined by one-way analysis of variance (ANOVA) followed by Duncan’s multiple range test for post hoc comparisons, conducted in SPSS Statistics 22.0 (SPSS Inc., Chicago, IL, USA). Significance levels are indicated as * *p* < 0.05, ** *p* < 0.01, and *** *p* < 0.001.

## 3. Results

### 3.1. Molecular Characterization of gFST Gene

**Figure 1:**
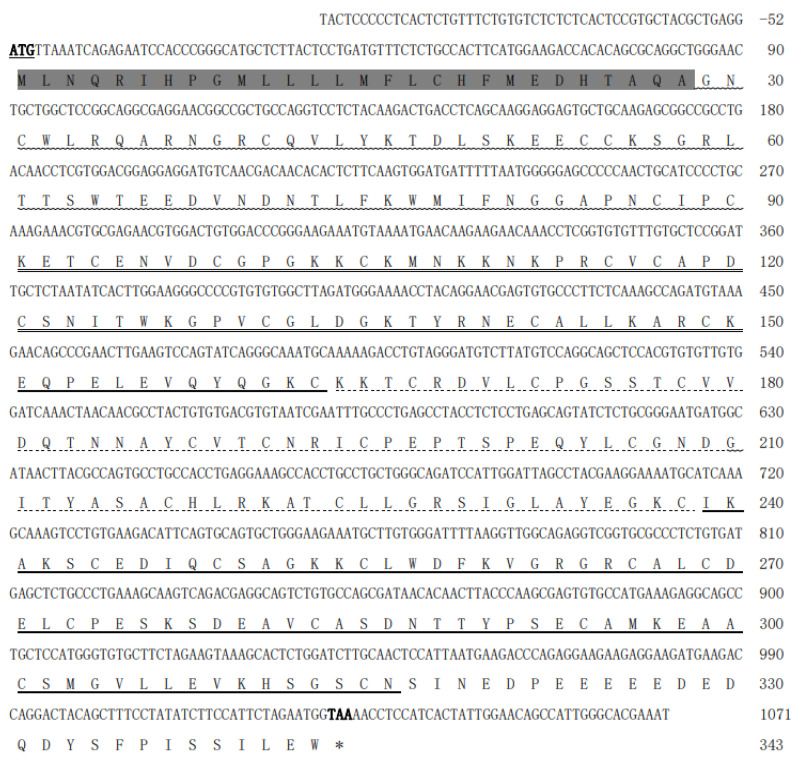
Nucleotide and deduced amino acid sequence of *gFST* gene (gFST-X1). The 1032 bp open reading frame encodes a 343-aa protein. The start codon (ATG) is bolded and underlined, and the stop codon (TAA) is marked by an asterisk *. The signal peptide is highlighted in gray. Conserved domains in the mature protein—N-domain (wavy underline), Domain I (double underline), Domain II (dotted underline), and Domain III (bold underline)—are indicated accordingly.

**Figure 2:**
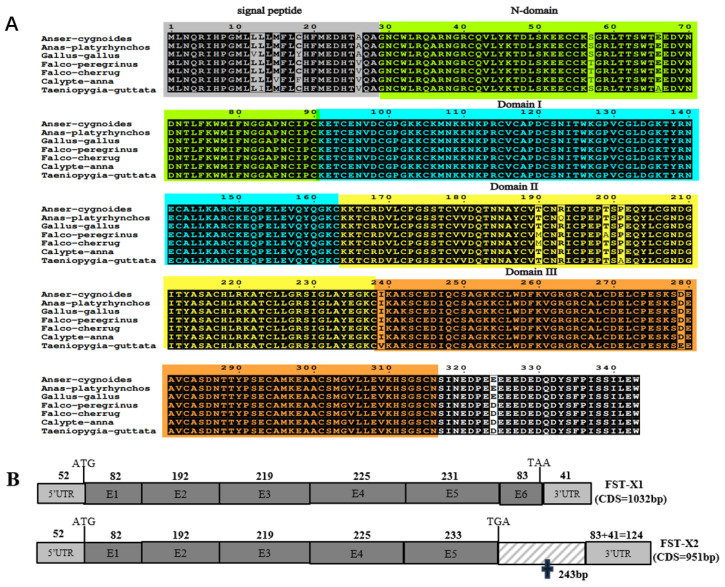
Sequence conservation and alternative splicing of *gFST* gene. (**A**) Multiple sequence alignment of FST proteins across avian species. Fully conserved residues are marked by asterisks; gaps are indicated by dashes. The signal peptide (gray), N-domain (green), Domain I (blue), Domain II (yellow), and Domain III (orange) are highlighted. (**B**) Schematic of two *gFST* splice variants. Exons (E1–E6) are shown as dark gray boxes, untranslated regions as light gray boxes, and deleted regions as white boxes. Nucleotide lengths are indicated above corresponding segments.

Sequence analysis demonstrated that the goose *FST* cDNA (gFST-X1, PQ165853) comprises 1125 nucleotides, including a 1032 bp open reading frame (ORF) flanked by 52 bp and 41 bp of the 5′- and 3′-untranslated regions, respectively, and terminating at a native polyadenylation site (Figure 1). Bioinformatics analysis revealed that the ORF was predicted to encode a 343 aa protein, which consists of a 28 aa signal peptide and a mature protein containing four conserved domains: an N-domain, Domain I, Domain II, and Domain III (Figure 1). Multiple sequence alignment demonstrated high conservation of the deduced FST protein among avian species, especially within Domain I and Domain III (Figure 2A).

Furthermore, we identified a transcript fragment exhibiting high sequence homology to gFST-X1, suggesting the existence of an alternative splicing variant of the *gFST* gene. This variant, designated gFST-X2 (PQ165854), contains a 951 bp ORF. Detailed sequence analysis demonstrated that gFST-X2 is generated by the insertion of a 243 bp fragment upstream of exon 6 in the gFST-X1 transcript. This insertion introduces a premature termination codon, resulting in the production of a truncated protein of 316 aa (Figure 2B).

### 3.2. Tissue Expression Pattern of gFST mRNA

**Figure 3:**
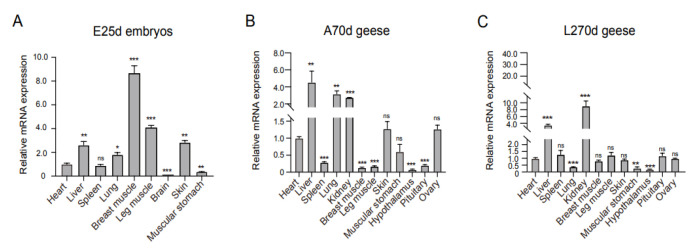
Expression profiles of *gFST* mRNA across three developmental stages in ZW geese. Tissue-specific expression patterns of *gFST* mRNA in (**A**) 25-day female embryos, (**B**) 70-day adult geese, and (**C**) 270-day laying geese. Relative expression levels were quantified by the comparative Ct method using *β-actin* as the reference gene (n = 4). Data are presented as mean ± SEM. Significance levels are indicated as * *p* < 0.05, ** *p* < 0.01, *** *p* < 0.001, and ns > 0.05.

Figure 3 displays the tissue-specific expression profiles of *gFST* mRNA in ZW geese at three developmental stages (E25d, A70d, and L270d) as quantified by qRT-PCR. In E25d embryos (Figure 3A), *gFST* expression was highest in breast and leg muscles (*p* < 0.001), moderate in the liver, skin, and lung (*p* < 0.01–0.05), and minimal in the muscular stomach and brain (*p* < 0.001). By A70d (Figure 3B), the highest expression levels were observed in the liver, lung, and kidney (*p* <0.001). Intermediate expression was detected in the heart, skin, ovary, and muscular stomach (*p* > 0.05), while significantly lower levels were found in the spleen, pituitary, breast muscle, leg muscle, and hypothalamus (*p* < 0.001). In L270d geese (Figure 3C), expression peaked in the kidney and liver (*p* < 0.001). Substantial expression was also detected in the heart, spleen, leg muscle, pituitary, ovary, breast muscle, and skin (*p* > 0.05), whereas significantly reduced expression was evident in the lung, muscular stomach, and hypothalamus (*p* < 0.001).

**Figure 4:**
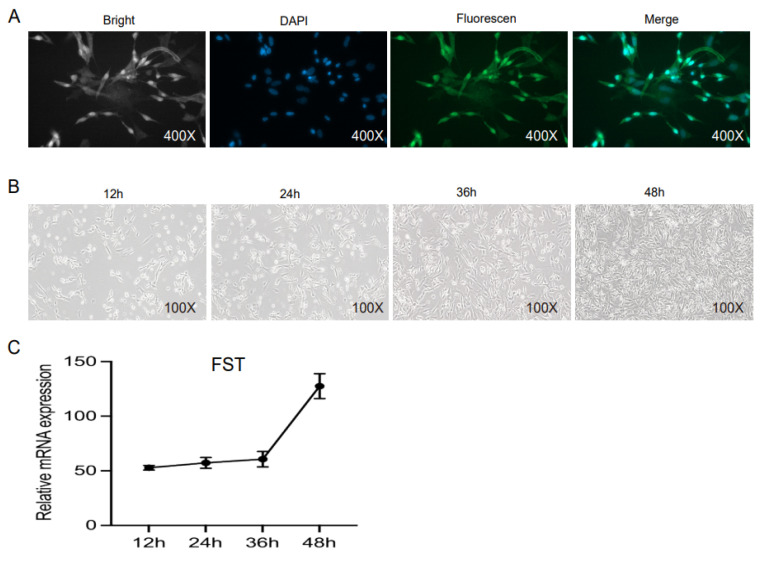
Identification, culture, and *FST* expression in goose SMSCs. (**A**) Immunofluorescence detection of *Pax7* in isolated SMSCs. (**B**) Morphology of SMSCs during 12–48 h of culture. Gray represents the brightfield, blue represents DAPI, green represents PAX7, and the combined (Gray + Blue + Green) represents Merge. (**C**) Relative *gFST* mRNA expression levels at different time points.

Given the high expression levels of *gFST* mRNA in the breast and leg muscle tissues of female E25d embryos (Figure 3A), and in light of our previous transcriptome data on goose SMSCs [22], we isolated and purified SMSCs from female E16d embryos to investigate the function of *gFST*. *Pax7*, a well-established specific marker for SMSCs, is widely used to differentiate SMSCs from other cell types. As shown in Figure 4A, immunofluorescence staining confirmed the expression of *Pax7* in the isolated cells, validating their identity as goose SMSCs.

Following isolation, the cells were cultured in growth medium and began to adhere within 12 h. By 24 h, they exhibited a dispersed growth pattern with approximately 90% adherence. Complete adherence was observed at 36 h, accompanied by a gradual elongation in cell morphology. The culture reached approximately 90% confluence after 48 h of incubation (Figure 4B). Concurrent qRT-PCR analysis revealed stable expression levels of gFST mRNA in the SMSCs across the 12, 24, 36, and 48 h time points (Figure 4C).

### 3.3. Gene Expression Analysis of FST-Overexpressed Cells

**Figure 5:**
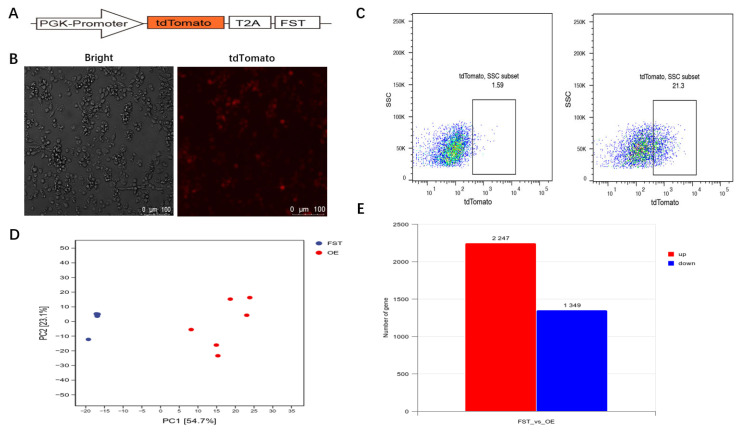
Transcriptome profiling of goose SMSCs following *gFST* overexpression (3-day culture). (**A**) Schematic representation of the *gFST* overexpression vector. (**B**) Microscopic image of transfected SMSCs. Greyscale: normal bright-field contrast; Red fluorescence: cells that have successfully taken the gFST-tdTomato construct; Scale bar: 100 μm. (**C**) Isolation of transfected cells by flow cytometry. Representative flow cytometry plots showing expression of tdTomato+ cells; The gated population (21.3%) represents tdTomato-positive cells. (**D**) PCA of transcriptome profiles from FST-OE and FST-WT groups (n = 6 per group). Red symbols: FST-OE (overexpression) replicates; Blue symbols: FST-WT (control) replicates. (**E**) Summary of DEGs between FST-OE and FST-WT groups (*p-adj* < 0.05). Red bar: number of up-regulated genes in FST-OE vs. WT; Blue bar: number of down-regulated genes in FST-OE vs. WT.

To functionally characterize *gFST* in SMSCs, an overexpression vector encoding the gFST-X1 transcript was constructed (Figure 5A) and transfected into SMSCs (Figure 5B). After three days of culture, transfected positive cells (21.3%) were isolated by FACS for subsequent RNA-seq analysis (Figure 5C). A total of 12 samples—six from the gFST-overexpressing population (FST-OE) and six from the wild-type control population (FST-WT)—were subjected to transcriptome sequencing. Principal component analysis (PCA) demonstrated clear separation between the transcriptomic profiles of the two groups, reflecting substantial differences in global gene expression (Figure 5D). Differential expression analysis identified 3596 significantly dysregulated genes (*p-adj* < 0.05), with 2247 upregulated and 1349 downregulated in the FST-OE group compared to FST-WT (Figure 5E). The complete list of DEGs is provided in Table S2, and all raw sequencing data have been deposited in the SRA database under accession number PRJNA1308531.

### 3.4. Identification of Key Genes Associated with SMSC Development

**Figure 6:**
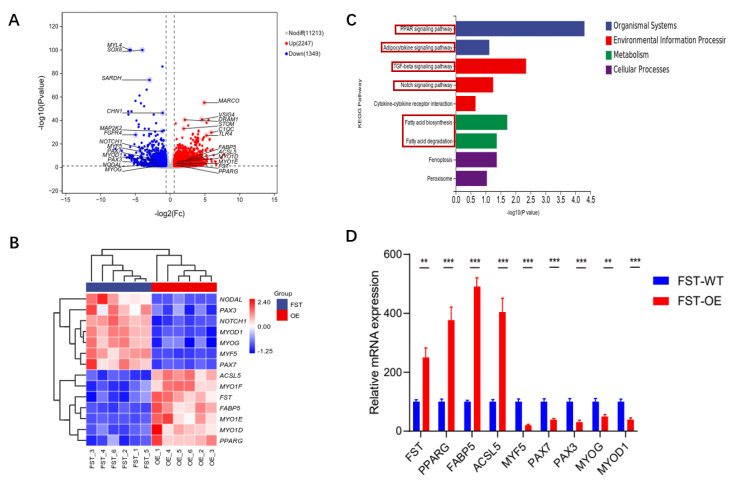
Analysis of DEGs in FST-overexpressing cells. (**A**) Volcano plot displaying up- and downregulated genes from transcriptome analysis. (**B**) Heatmap of DEGs associated with muscle development. (**C**) KEGG pathways enrichment analysis of DEGs. The PPAR signaling, TGF-β signaling, fatty-acid metabolism, and Notch signaling pathways are highlighted with red frames. (**D**) qRT-PCR validation of selected DEGs. Gene expression was normalized to *β-actin* and shown as mean ± SEM (n = 6). Significance levels: ** *p* < 0.01, *** *p* < 0.001.

The *FST* gene acts as a critical regulator of skeletal muscle growth and fat deposition, playing a key role in multiple metabolic pathways involved in myogenesis. As depicted in the volcano plot in Figure 6A, overexpression of *gFST* in SMSCs led to significant downregulation of key marker genes, including *PAX7* (*p-adj* = 1.71 × 10^−6^) and *PAX3* (*p-adj* = 0.00191). Similarly, essential myogenic regulatory factors such as *MYOG* (*p-adj* = 0.034659), *MYOD1* (*p-adj* = 1.62 × 10^−5^), *MYF5* (*p-adj* = 1.62 × 10^−12^), *NOTCH1* (*p-adj* = 8.53 × 10^−11^), and *NODAL* (*p-adj* = 0.02484) were also suppressed. In contrast, *gFST* overexpression markedly upregulated genes associated with lipid metabolism, including *PPARG* (*p-adj* = 0.044521), *FABP5* (*p-adj* = 1.62 × 10^−6^), and *ACSL5* (*p-adj* = 2.00 × 10^−5^) as well as members of the myosin-I family: *MYO1D* (*p-adj* = 9.81 × 10^−5^), *MYO1F* (*p-adj* = 3.88 × 10^−6^), and *MYO1E* (*p-adj* = 0.000225) (Figure 6A). The expression patterns of these key DEGs are displayed in Figure 6B.

KEGG pathway analysis indicated that the DEGs were enriched in 251 pathways, 39 of which were significantly enriched (*p-adj* < 0.05) (Table S3). Notably, the key DEGs described above were primarily associated with the PPAR signaling pathway, TGF-beta signaling pathway, Notch signaling pathway and fatty acid metabolism (Figure 6C). Eight DEGs were further validated by qRT-PCR, which showed a strong and statistically significant correlation with the RNA-seq data (*p* < 0.01, Figure 6D).

## 4. Discussion

The *FST* gene, a pivotal member of the TGF-β superfamily, is characterized by a conserved genomic structure across species, typically spanning approximately 6 kilobases and comprising six exons. This structure facilitates the generation of multiple isoforms through alternative splicing. In mammals, the primary isoforms are FST-317 and FST-344 [37,38], while in birds such as chickens and ducks, the FST-343 isoform has been identified [11,13]. In this study, we identified both the FST-343 transcript and a novel shorter variant (FST-316) in geese, which results from alternative splicing. This discovery suggests that the alternative splicing landscape of the *FST* gene in geese is more complex than previously recognized, and the functional implications of these distinct isoforms warrant further investigation.

The FST protein plays a crucial role not only in muscle growth and bone metabolism but also in the regulation of various biological processes—including the development of vital organs, reproduction, hair follicle morphogenesis, and red blood cell regeneration—during embryonic and postnatal development in mammals and birds [39]. Its expression has been documented in diverse tissues across species, including skeletal muscle, heart, liver, and kidney in pigs [40], as well as in embryonic somites and post-hatch reproductive and circulatory tissues in chickens [13]. Our results further demonstrate that *FST* is widely expressed in geese throughout development, exhibiting dynamic spatiotemporal patterns. At the embryonic stage (E25d), expression was highest in breast muscle, leg muscle, liver, and skin, indicating its involvement in early muscle and organ development. By the post-hatch stage (A70d), elevated expression was observed in liver, lung, kidney, ovary, and heart, suggesting a functional shift toward metabolic and reproductive processes. In the laying stage (L270d), expression became strongly localized to the kidney and liver, underscoring a sustained role in metabolic regulation and reproductive physiology. These findings confirm that *FST* is expressed across a wide range of tissues and participates in stage-specific physiological pathways throughout development.

The genetic regulation of muscle development in poultry is complex. Transcriptomic studies in chickens, ducks, and geese have begun to unravel the gene networks and signaling pathways controlling myogenesis [41,42,43,44,45,46,47,48]. Building on this foundation, our functional investigation demonstrates that *FST* overexpression in goose SMSCs induces a profound transcriptomic shift. This shift is defined by the coordinated downregulation of core myogenic regulators—including stemness factors (*PAX7*, *PAX3*), determination genes (*MYOD*, *MYF5*), and the terminal differentiation marker *MYOG*—as well as key signaling components (*NOTCH1*, *NODAL*). This pattern indicates a departure from the canonical myogenic differentiation program.

Concurrently, we observed a marked upregulation of genes associated with lipid metabolism (*PPARG*, *FABP5*, *ACSL5*) and cytoskeletal organization (*MYO1D*, *MYO1F*, *MYO1E*). This suggests that FST not only modulates myogenic commitment but also promotes a state of enhanced metabolic capacity and structural maturation. The KEGG pathway analysis further substantiates these findings, showing significant enrichment in the PPAR signaling pathway, fatty acid metabolism, and the TGF-β and Notch signaling pathways.

While previous research in zebrafish and mice has established that FST promotes muscle growth primarily through cellular hyperplasia [49,50], our data reveal a more complex, multi-faceted role in geese. We propose that FST enhances skeletal muscle development through a dual mechanism: stimulating cellular proliferation while simultaneously redirecting SMSCs toward a developmental trajectory characterized by metabolic enrichment and cytoarchitectural specialization. This combined action likely contributes to improvements in both muscle mass and meat quality.

## 5. Conclusions

In conclusion, we identified two novel transcript variants of the goose *FST* gene. Our study provides crucial insights into the molecular mechanisms by which *FST* influences muscle development and meat quality traits in geese. Future research should prioritize lineage-tracing studies to delineate the fate of FST-overexpressing SMSCs, functional validation of the implicated metabolic pathways, and comparative analyses across different goose breeds. These efforts will be instrumental for advancing genetic selection strategies aimed at enhancing meat quality.

## Data Availability

The datasets utilized in this study are publicly accessible through online repositories. Specifically, the transcriptome data are deposited in the NCBI BioProject database under accession number PRJNA1308531 (https://www.ncbi.nlm.nih.gov/bioproject/PRJNA1308531, accessed on 20 August 2025).

## Supplementary Materials

The following supporting information can be downloaded at: https://www.mdpi.com/article/10.3390/ani15203009/s1. Table S1: Primers used in this study. Table S2: The complete list of DEGs. Table S3: The complete list of KEEG Ppathways. The datasets utilized in this study are publicly accessible through online repositories. Specifically, the transcriptome data are deposited in the NCBI BioProject database under accession number PRJNA1308531 (https://www.ncbi.nlm.nih.gov/bioproject/PRJNA1308531, accessed on 20 August 2025).
