# Supplementary material for: Comparative Transcriptome Analysis Reveals the Role of the FST Gene in Goose Muscle Development
Source: Animals (Basel). 2025 Oct 16;15(20):3009. doi: 10.3390/ani15203009 (PMC12560870; doi:10.3390/ani15203009)
Supplement: Supplementary file 1 [file animals-15-03009-s001.zip › Table S1.pdf]

Table S1 Primers used in this study

Purpose	Name	Sequence (5’→3’)	Accession number	Size(bp)	Tm (°C)
Sex test	CHD1-F	TGCAGAAGCAATATTACAAGT	NC_089912.1	466/326	60
	CHD1-R	AATTCATTATCATCTGGTGG			
RT-PCR	FST-F	TACTCCCCCTCACTCTGTTTCT	XM_013180613.3	1368/1125	60
	FST-R	TGATTTCGTGCCCAATGGCTGT			
Expression profile	FST-QF	CACCATCAGGAACATCACCAA	PQ165853	208	60
	FST-QR	GTTATGGGCCAAGATTTTCAG			
Internal control	β-actin-F	TCCGTGACATCAAGGAGAAG	M26111.1	224	60
	β-actin-R	CATGATGGAGTTGAAGGTGG			
qRT-PCR validation	PPARG-F	CAGGAGCAGAACAAAGAGGTA	XM_067003362.1	185	60
	PPARG-R	GAAGCCAGGAGAGTATATATGA			
	ACSL5-F	GGAAAGACCCCATGTGTGAAGA	XM_013174554.3	175	60
	ACSL5-R	ACACAATGCAAAGATCTTCAGG			
	FABP5-F	ACAATCACCGTAAAAACAGAAA	XM_048068394.2	186	60
	FABP5-R	AAGTTTCCGTGTTATTATGGTC			
	MYF5-F	CACCAGCCTACCGAGCCCGCC	XM_048078888.2	203	60
	MYF5-R	CTCTTCAGGGTCTCAAACGCC			
	PAX3-F	AGAAAGCAGCGTCGTAGCAG	KT380623.1	275	60
	PAX3-R	GAGAGCTGGTATGTCGGCAA			
	PAX7-F	CCCAAGATGGGGGAAGCACT	KY381597.1	190	60
	PAX7-R	TTGCTAACAGGATTCATGTG			
	MYOG-F	GCACTGCCCCGGGCAATGCC	KT290042.1	200	60
	MYOG-R	TGGCGCTGCGCAGGATCTCC			
	MYOD1-F	CGGCTCAGCAAGGTCAACGA	XM_013177726.3	210	60
	MYOD1-R	GTTGGAGCGGGGGCTGGAGG			
